# Neurostimulant Use and Cognitive Outcomes in Patients with Acute, Severe Traumatic Brain Injury

**DOI:** 10.1007/s12028-025-02400-3

**Published:** 2025-11-05

**Authors:** Brett Tracy, Shruthi Srinivas, Kelly Nahum, Russell Payne, Jacob Roden-Foreman, Anna Liveris, Joy Song, Michelle Kincaid, Stephanie Doris, William Brigode, Johanna Stecher, Tanya Egodage, Anthony Tigano, Kaushik Mukherjee, Liz Penaloza-Villalobos, Katherine McBride

**Affiliations:** 1 The Ohio State University Wexner Medical Center, Columbus, OH USA; 2 Montefiore Medical Center, Bronx, NY USA; 3 Texas Health Presbyterian Hospital, Dallas, TX USA; 4 NYC Health + Hospitals/Jacobi Medical Center, Bronx, NY USA; 5 Ohio Health Grant Medical Center, Columbus, OH USA; 6 Cook County Hospital, Chicago, IL USA; 7 Cooper University Hospital, Camden, NJ USA; 8 Loma Linda University Medical Center, Loma Linda, CA USA; 9 Memorial Health University Medical Center, Savannah, GA USA

**Keywords:** Neurostimulants, Traumatic brain injury, Disorders of consciousness, Cognitive function, Disability rating scale

## Abstract

**Background:**

We sought to examine the use of neurostimulants (NS) in the acute setting among patients with severe traumatic brain injury (TBI) and assess their impact on cognitive outcomes.

**Methods:**

We performed a retrospective analysis of prospectively collected data from patients aged ≥ 18 years with a severe (Glasgow Coma Scale score ≤ 8), blunt TBI at eight US trauma centers from 2020 to 2023. Patients were grouped according to whether they received NS. Our primary outcome was cognitive disability at 28 days or discharge (whichever occurred first), measured by the Disability Rating Scale (DRS). A DRS score < 12 was considered a favorable outcome.

**Results:**

The cohort comprised 405 patients: 29.9% (*n* = 121) received NS and 70.1% (*n* = 284) did not. The most common NS was amantadine (97.5%, *n* = 118). The median time to NS initiation was 8.0 days (5.0–13.5), and the median treatment duration was 12.0 days (5.5–17.0). NS patients had worse injury severity scores (29 vs. 26, *p* = 0.02), had more neurosurgical interventions (64.4% vs. 33.2%, *p* < 0.0001), and were less likely to have a favorable outcome (30.8% vs. 67.1%, *p* < 0.0001). In a subgroup analysis of only NS patients, individuals receiving NS within 7 days of admission were three times as likely to have a favorable outcome compared to those receiving NS > 7 days after admission (odds ratio 3.01, 95% confidence interval 1.11–8.18, *p* = 0.03).

**Conclusions:**

Nearly a third of patients with severe TBI received NS. Patients receiving NS were more injured and had worse disability at discharge compared to non-NS patients. However, among patients who received NS, treatment within 7 days was associated with an increased odds of a favorable outcome.

**Supplementary Information:**

The online version contains supplementary material available at 10.1007/s12028-025-02400-3.

## Introduction

In the United States, nearly 1.7 million individuals sustain a traumatic brain injury (TBI) each year [1]. Although most of these injuries are mild and resolve without consequence, some patients experience profound brain damage and become clinically obtunded [1]. This spectrum of diminished consciousness, commonly referred to as a disorder of consciousness (DoC), includes coma, unresponsive wakefulness syndrome (UWS), or minimally conscious state [2]. Only 50% of patients with UWS regain consciousness at 1 year, and roughly 50% of minimally conscious patients remain disabled at 1 year [3]. Even for individuals who have emerged from a DoC, approximately one third still have a compromised quality of life due to financial, cognitive, or psychiatric reasons [4].

Fortunately, there is increasing evidence to suggest that cognitive impairment after TBI can be improved with neurostimulants (NS) [1, 5]. Amantadine is the most commonly used NS to treat patients with impaired consciousness. It has been shown to accelerate return to consciousness and cognitively mediated behaviors for patients with UWS or in a minimally conscious state during the TBI rehabilitation phase [3, 6, 7]. Methylphenidate, a central nervous system NS, inhibits dopamine and norepinephrine transport and reuptake [8, 9]. Its use in the postacute recovery phase is associated with improved cognitive functioning speeds among patients with moderate to severe TBI [10]. Modafinil is another NS that acts on both the dopaminergic and noradrenergic pathways. Its use is also linked to improved consciousness and cognition in patients with a DoC [11–13].

Given the natural trajectory of recovery after TBI, most NS studies assess cognitive outcomes several months post injury. Less is known about the impact of NS on acute outcomes, which even if not indicative of ultimate recovery, could still offer insight into the benefits of NS. The purpose of this study is to examine NS prescribing patterns in the acute setting at several trauma centers. We will also assess the impact of NS timing on acute cognitive impairment and hypothesize that earlier exposure to NS will be associated with a greater improvement in cognitive outcomes.

## Methods

We performed a retrospective analysis of prospectively collected data between 2020 and 2023 on patients with severe, blunt TBI. Eight academic trauma centers participated in the study. Each site obtained their own institutional review board approval and provided waivers of informed consent. The Eastern Association for the Surgery of Trauma Multicenter Trials Committee also approved this study.

### Patients

We included patients aged > 18 years who presented with a Glasgow Coma Scale (GCS) score of ≤ 8 and with radiographic evidence of an intracranial injury. Patients who died or were discharged within 48 h of arrival were excluded. Prisoners, pregnant patients, patients with penetrating trauma, and individuals with cognitive disability predating the TBI were excluded. We considered cognitive impairment as an inability to interact or communicate, history of a neurologic disorder (i.e., dementia, developmental delay), schizophrenia, or psychosis. We also excluded patients with known seizure disorder, prior treatment with an NS within 30 days, Parkinson disease, and an allergy or a previous adverse reaction to an NS.

The entire cohort was divided into groups based on whether they received an NS within the first 28 days of their acute hospitalization. The decision to prescribe an NS was at the discretion of the intensivist. No clinical interventions were performed for the purpose of the study, and patients received treatment as deemed appropriate by the critical care team at each institution.

### Data Points

We collected data on patient age, demographics, and Charlson comorbidity index (CCI). Mechanism of injury, injury severity score (ISS), presenting GCS score, and radiographic TBI findings on computed tomography (CT) and/or magnetic resonance imaging (MRI) were recorded. Radiographic findings were categorized by Marshall scores (I–VI) as previously defined [14, 15]. Additional intracranial pathology not specifically included in the Marshall score (subarachnoid hemorrhage, subdural hemorrhage, intraventricular hematoma, epidural hematoma, diffuse axonal injury [DAI]) was collected [16]. Neurosurgical interventions (NSIs; craniotomy and/or craniectomy), propranolol and antipsychotic use, tracheostomy tube placement, and percutaneous endoscopic gastrostomy (PEG) tube placement were tracked. Propranolol data were collected because propranolol is believed to mitigate sympathetic hyperactivity following TBI, correlates with better cognitive outcomes, and decreases mortality [17, 18]. Antipsychotic use was tracked because these medications can directly target dopamine receptors, which can alter the effect of NS [19, 20].

For patients who received NS, the type, time to initiation, and duration of treatment were recorded. For the purposes of this study, the NS tracked were amantadine, methylphenidate, and modafinil because they are the most frequently used and studied in the acute and postacute setting [21–23]. Variation in prescribing patterns between the eight sites was also recorded.

We also collected Disability Rating Scale (DRS) scores and GCS scores at 28 days or discharge (whichever occurred first), which we will refer to as final DRS and GCS scores, respectively. We chose the DRS because it has better precision than the Glasgow Outcome Scale and can track an individual from coma to community [24]. The DRS score can also be performed and scored through the person or by proxy and is more sensitive to subtle functionally limiting cognitive deficits [25]. The DRS assesses cognitive and functional impairment, disability, and handicap and yields a score from 0 to 29, with higher scores being worse. It can also be binned into impairment categories [3, 26]. For this study, we defined DRS scores < 12 as a favorable outcome and DRS scores ≥ 12 as unfavorable outcome.

### Outcomes

Our primary study outcome was the proportion of patients with a favorable outcome compared between patients receiving NS and patients not receiving NS. Secondary outcomes included final DRS scores (continuous), final GCS scores, hospital length of stay (LOS), intensive care unit (ICU) LOS, duration of mechanical ventilation, in-hospital adverse events, and medication-specific adverse events. In-hospital adverse events included acute kidney injury (AKI), bacteremia, cardiopulmonary arrest, pneumonia, ICU readmission, venous thromboembolism (VTE), and urinary tract infection (Appendix A). Medication-specific adverse events related to NS included tachyarrhythmias and seizures. Additional secondary outcomes were in-hospital mortality/hospice and discharge disposition (home, long-term acute care hospital [LTACH]/skilled nursing facility [SNF], and inpatient rehabilitation [IPR]).

We also performed a predetermined subgroup analysis evaluating only patients who received NS. These patients were divided into two groups based on the timing of initiation of NS therapy: early (≤ 7 days) or late (> 7 days). We assessed the same outcomes as in our primary analysis.

### Data Management

Data were entered as case report forms into Research Electronic Data Capture (REDCap) [27]. Each participating site was given access to REDCap, which was housed at the host institution. No protected health information was entered into the database to limit the risk of breach of confidentiality. Data entries were audited and validated by the research team.

### Statistical Analysis

Based on previous pilot study data, we estimated a sample of 376 patients to detect a 30% difference in the proportion of our cognitive impairment outcome (alpha = 5%, power = 80%) [28]. Continuous variables between the eight study sites were compared using analysis of variance tests with a Bonferroni correction. To identify variables that were associated with NS, continuous variables were compared between groups using Mann–Whitney *U*-tests (nonparametric distribution) and are presented as medians (interquartile range [IQR]). Categorical data were compared with Pearson χ^2^ tests or Fisher’s exact tests when appropriate and are presented as counts (percentages). All patients were included in the analysis except when evaluating cognitive impairment, final DRS scores, and final GCS scores. These specific outcomes were analyzed after excluding patients who died or who were discharged within 7 days of admission. Including only patients hospitalized for more than 7 days in this particular outcome analysis more appropriately reflected an LOS consistent with severe TBI [29–31].

The same analyses and outcomes used for the overall cohort were performed for our subgroup analysis comparing patients who received early and late NS. We also sought to identify predictors of a favorable outcome among NS patients using backward stepwise logistic regression. The following variables chosen a priori were entered into the stepwise regression model: age group, Marshall score, additional intracranial pathology, ISS, ICU LOS, and NS timing (early vs. late). To create a more clinically interpretable model and because there were no Marshall scores of VI in the subgroup, scores were dichotomized into surgically evacuated lesions (V) versus nonevacuated lesions (I–IV). Our final multivariable regression model was then assessed for predictive ability using the area under the receiver operating characteristic curve (AUROC). A *p* value of less than 0.05 was considered statistically significant. Data analyses were performed with JMP version 18.2 (SAS Institute Inc., Cary, NC).

## Results

### Cohort Characteristics

The cohort comprised 405 patients: 29.9% (*n* = 121) received NS and 70.1% (*n* = 284) did not. Most patients presented after a motor vehicle collision (47.9%, n = 194), with a median initial GCS score of 3 (IQR 3–6) and a Marshall score of II (42.2%, *n* = 171). There was no difference in age group (*p* = 0.4770), sex (*p* = 0.3291), race (*p* = 0.8655), or CCI categories between the two patient groups (Table 1). The Abbreviated Injury Scale-Head scores were similar (*p* = 0.2359), but ISS scores were greater in the NS group (29 vs. 26, *p* = 0.0208). More patients in the NS group had DAI (40.5% vs. 19.0%, *p* < 0.0001), received propranolol (56.2% vs. 10.9%, *p* < 0.0001), and were more likely to undergo an NSI (62.8% vs. 40.1%, *p* < 0.0001), PEG tube placement (65.3% vs. 22.9%, *p* < 0.0001), and tracheostomy tube placement (63.6% vs. 23.6%, *p* < 0.0001).

**Table 1:** Patient characteristics (*N* = 405)

	No NS (*n* = 284)	NS (*n* = 121)	*p*
*Age group, n (%)*			
18–35 y	111 (39.1)	45 (37.2)	0.4770
36–55 y	69 (24.3)	38 (31.4)	
56–75 y	79 (27.8)	30 (24.8)	
≥ 76 y	25 (8.8)	8 (6.6)	
Male sex, *n* (%)	210 (73.9)	95 (78.5)	0.3291
*Race, n (%)*			
Asian	7 (2.5)	3 (2.5)	
Black	59 (20.8)	29 (24.0)	
Hispanic	56 (19.7)	26 (21.5)	0.8655
Other	7 (2.5)	1 (0.8)	
Unknown	14 (4.9)	6 (5.0)	
White	141 (49.6)	56 (46.3)	
*Charlson comorbidity index, n (%)*			
0	220 (77.5)	105 (86.8)	
1	15 (5.3)	3 (2.5)	0.1898
2	30 (10.6)	8 (6.6)	
≥ 3	19 (6.7)	5 (4.1)	
*Injury mechanism, n (%)*			
Assault	22 (7.7)	7 (5.8)	
Fall > 8 feet	30 (10.6)	8 (6.6)	
Found down	10 (3.5)	2 (1.7)	0.1909
Ground level fall	47 (16.5)	13 (10.7)	
Motor vehicle collision	126 (44.4)	68 (56.2)	
Pedestrian struck	49 (17.3)	23 (19.0)	
Index GCS score, median (IQR)	3 (3–6)	3 (3–5)	0.1201
Injury severity score, median (IQR)	26 (18–30)	29 (22–38)	0.0208
AIS head score, median (IQR)	4 (3–5)	4 (3–5)	0.2359
*Marshall score, n (%)*			
I	39 (13.7)	10 (8.3)	
II	118 (41.5)	53 (43.8)	0.2130
III	31 (10.9)	12 (9.9)	
IV	23 (8.1)	9 (7.4)	
V	66 (23.2)	37 (30.6)	
VI	7 (2.5)	0 (0.0)	
*Intracranial pathology, n (%)*			
Diffuse axonal injury	54 (19.0)	49 (40.5)	< 0.0001
Epidural hematoma	20 (7.0)	8 (6.6)	0.8757
Intraventricular hemorrhage	20 (7.0)	14 (11.6)	0.0134
Subarachnoid hemorrhage	212 (74.6)	89 (73.6)	0.8175
Subdural hemorrhage	106 (37.3)	58 (47.9)	0.05
Neurosurgical intervention,* n* (%)	114 (40.1)	76 (62.8)	< 0.0001
Propranolol,* n* (%)	31 (10.9)	68 (56.2)	< 0.0001
Antipsychotics,* n* (%)	120 (42.3)	58 (47.9)	0.2918
PEG tube,* n* (%)	65 (22.9)	79 (65.3)	< 0.0001
Tracheostomy,* n* (%)	67 (23.6)	77 (63.6)	< 0.0001

AIS, abbreviated injury scale; GCS, Glasgow Coma Scale; IQR, interquartile range; NS, neurostimulant; PEG, percutaneous endoscopic gastrostomy

The most common NS prescribed was amantadine (97.5%, *n* = 118), followed by methylphenidate (9.1%, *n* = 11) and modafinil (3.1%, *n* = 4). The median time to NS initiation was 8.0 days (IQR 5.0–13.5), and the median therapy duration was 12.0 days (IQR 5.5–17.0). There was significant variation between centers with respect to NS prescribing frequency (*p* = 0.0001) (Appendix B); however, there was no difference in time to starting NS between sites (*p* = 0.1598) or in overall duration of therapy (*p* = 0.4481).

### Study Outcomes

After we excluded patients who died or were discharged before 7 days (*n* = 128), patients in the NS group were less likely to have a favorable outcome (30.8% vs. 67.1%, *p* < 0.0001) and had worse final DRS scores (18 vs. 6, *p* < 0.0001) (Table 2). Among all patients in the cohort (*N* = 405), rates of AKI (13.2% vs. 7.0%, *p* = 0.0454), bacteremia (12.4% vs. 3.2%, *p* = 0.0008), pneumonia (49.6% vs. 22.5%, *p* < 0.0001), and VTE (12.4% vs. 4.6%, *p* = 0.0088) were higher in the NS group. Hospital LOS, ICU LOS, and mechanical ventilation duration were significantly longer in the NS group, whereas hospital, ICU, and mechanical ventilation free days were shorter. Discharge disposition varied between groups (*p* < 0.0001) (Table 2). On pairwise comparison, mortality (12.4% vs. 28.9%, *p* = 0.0003) and home discharge (7.4% vs. 19.0%, *p* = 0.0026) rates were lower, whereas IPR (47.9% vs. 35.6%, *p* = 0.026) and LTACH/SNF (29.8% vs. 13.4%, *p* = 0.0002) discharges were higher in the NS group.

**Table 2:** Study outcomes (*N* = 405)

	No NS (*n* = 284)	NS (*n* = 121)	*p*
*Cognitive outcome, n (%)*^*a*^			
Favorable	116 (67.1)	32 (30.8)	< 0.0001
Unfavorable	57 (32.9)	72 (69.2)	
Final DRS score, median (IQR)^a^	6 (2–15)	18 (10–24)	< 0.0001
Final GCS score, median (IQR)^a^	14 (11–15)	11 (8–15)	0.0791
Hospital LOS, median (IQR), d	15 (6–25)	28 (18–44)	< 0.0001
ICU LOS, median (IQR), d	8 (4–17)	18 (11–28)	< 0.0001
Mechanical ventilation duration, median (IQR), d	6 (3–12)	13 (8–20)	< 0.0001
Hospital free days, median (IQR)^b^	13 (3–22)	0 (0–10)	< 0.0001
ICU free days, median (IQR)^b^	20 (11–24)	10 (0–17)	< 0.0001
Mechanical ventilation free days, median (IQR)^b^	22 (16–25)	15 (8–20)	< 0.0001
*Medication-specific adverse events, n (%)*			
Seizures	13 (4.6)	8 (6.6)	0.4631
Tachyarrhythmia	14 (4.9)	4 (3.3)	0.6028
Inpatient adverse events, *n* (%)			
Acute kidney injury	20 (7.0)	16 (13.2)	0.0454
Bacteremia	9 (3.2)	15 (12.4)	0.0008
Cardiopulmonary arrest	12 (4.2)	9 (7.4)	0.2204
ICU readmission	9 (3.2)	8 (6.6)	0.1724
Pneumonia	64 (22.5)	60 (49.6)	< 0.0001
Urinary tract infection	1 (0.4)	5 (4.6)	0.0130
Venous thromboembolism	13 (4.6)	15 (12.4)	0.0088
*Discharge disposition, n (%)*			
Dead/hospice	82 (28.9)	15 (12.4)	< 0.0001
Home	54 (19.0)	9 (7.4)	
Inpatient rehabilitation	101 (35.6)	58 (47.9)	
LTACH/SNF	38 (13.4)	36 (28.9)	
Unknown/other	9 (3.2)	3 (2.5)	

DRS, Disability Rating Scale; GCS, Glasgow Coma Scale; ICU, intensive care unit; IQR, interquartile range; LOS, length of stay; LTACH, long-term acute care hospital; NS, neurostimulant; SNF, skilled nursing facility

^a^Excludes patients (*n* = 128) with a hospital LOS < 7 days or who died/were discharged to hospice

^b^Days free within 28 days

### NS Subgroup Characteristics

We then performed a subgroup analysis of patients only receiving NS (*n* = 121): 43% (*n* = 52) received early NS and 57% (*n* = 69) received late NS. Patient demographics, injury mechanism and scores, and TBI pathology were similar between groups (Table 3). However, patients in the early group were less likely to undergo an NSI (51.9% vs. 71.0%, *p* = 0.0315), PEG tube placement (42.3% vs. 82.6%, *p* < 0.0001), and tracheostomy tube placement (46.2% vs. 76.8%, *p* = 0.0005). There was no difference in duration of NS therapy between groups (10.0 vs. 12.0 days, *p* = 0.4472) (Table 3).

**Table 3:** Characteristics of patients receiving NS (*n* = 121)

	Early (*n* = 52)	Late (*n* = 69)	*p*
*Age group, n (%)*			
18–35 y	20 (38.5)	25 (36.2)	0.4502
36–55 y	13 (25.0)	25 (36.2)	
56–75 y	14 (26.9)	16 (23.2)	
≥ 76 y	5 (9.6)	3 (4.3)	
Male sex, *n* (%)	39 (75.0)	56 (81.2)	0.4142
*Race, n (%)*			
Asian	1 (1.9)	2 (2.9)	0.6008
Black	12 (23.1)	17 (24.6)	
Hispanic	9 (17.3)	17 (24.6)	
Other	1 (1.9)	0 (0.0)	
Unknown	4 (7.7)	2 (2.9)	
White	25 (48.1)	31 (44.9)	
*Charlson comorbidity index, n (%)*			
0	44 (84.6)	61 (88.4)	0.8217
1	2 (3.8)	1 (1.4)	
2	4 (7.7)	4 (5.8)	
≥ 3	2 (3.8)	3 (4.3)	
*Injury mechanism, n (%)*			
Assault	1 (1.9)	6 (8.7)	
Fall > 8 feet	6 (11.5)	2 (2.9)	
Found down	1 (1.9)	1 (1.4)	0.2468
Ground level fall	7 (13.5)	6 (8.7)	
Motor vehicle collision	27 (51.9)	41 (59.4)	
Pedestrian struck	10 (19.2)	13 (18.8)	
Index GCS score, median (IQR)	3 (3–6.75)	3 (3–4)	0.1852
Injury severity score, median (IQR)	28 (18.25–34)	30 (22–38)	0.2287
AIS head score, median (IQR)	4 (3–5)	4 (3–5)	0.6624
*Marshall score, n (%)*			
I	3 (5.8)	7 (10.1)	0.2641
II	27 (51.9)	26 (37.7)	
III	7 (13.5)	5 (7.2)	
IV	3 (5.8)	6 (8.7)	
V	12 (23.1)	25 (36.2)	
*Intracranial pathology, n (%)*			
Diffuse axonal injury	17 (32.7)	32 (46.4)	0.1290
Epidural hematoma	4 (7.7)	4 (5.8)	0.6779
Intraventricular hemorrhage	6 (11.5)	8 (11.6)	0.9924
Subarachnoid hemorrhage	36 (69.2)	53 (76.8)	0.8761
Subdural hemorrhage	18 (45.0)	33 (51.6)	0.5149
Neurosurgical intervention, *n* (%)	27 (51.9)	49 (71.0)	0.0315
Propranolol, *n* (%)	26 (50.0)	42 (60.9)	0.2329
Antipsychotics, *n* (%)	24 (46.2)	34 (49.3)	0.7337
PEG tube, *n* (%)	22 (42.3)	57 (82.6)	< 0.0001
Tracheostomy, *n* (%)	24 (46.2)	53 (76.8)	0.0005
*NS timing, median (IQR), d*			
Time to initiation	5 (3–6.75)	13 (9–17)	< 0.0001
Duration of therapy	10 (4.25–21)	12 (7–15.5)	0.4472

AIS, abbreviated injury scale; GCS, Glasgow Coma Scale; IQR, interquartile range; NS, neurostimulant; PEG, percutaneous endoscopic gastrostomy

### NS Subgroup Outcomes

**Fig. 1:**
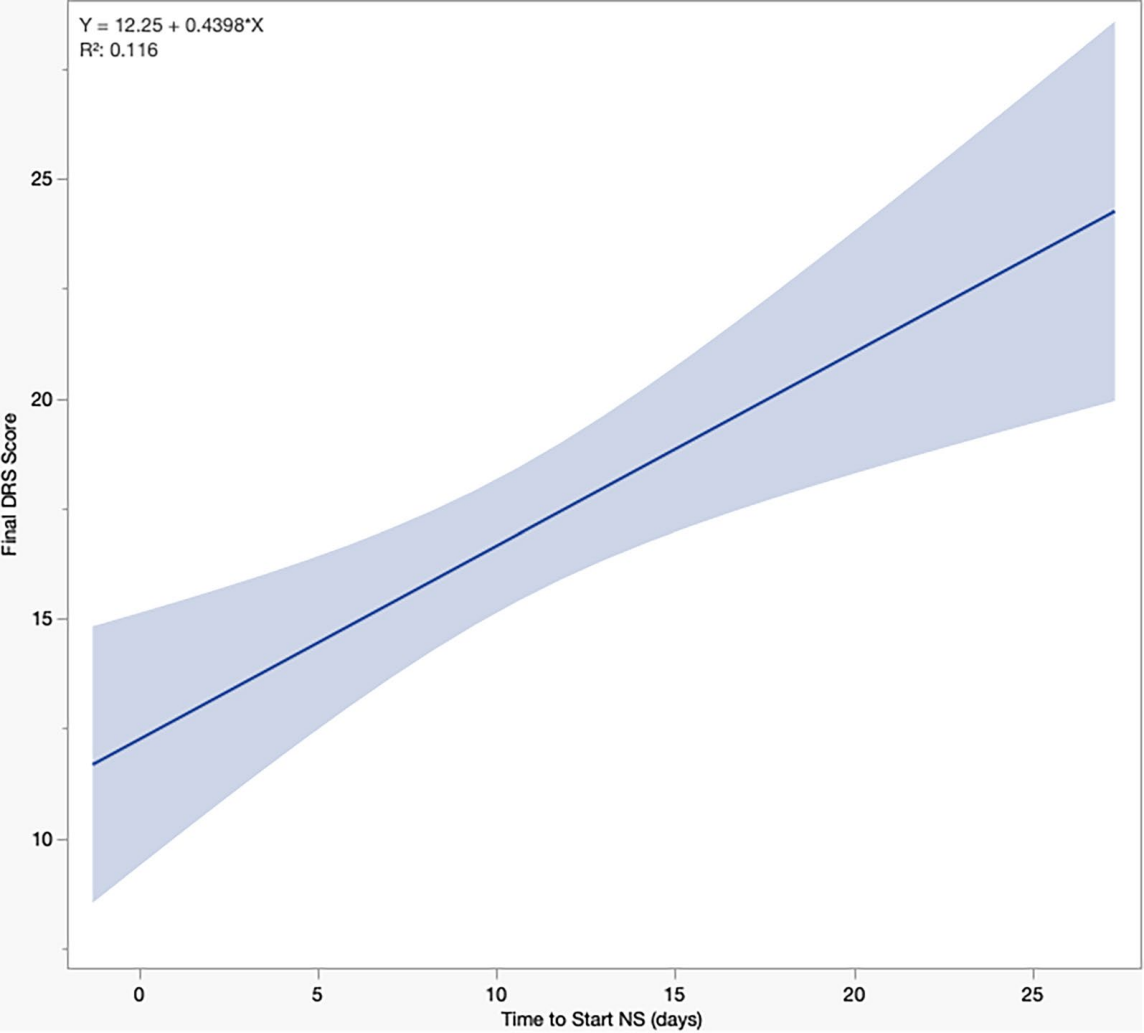
Relationship between time to neurostimulant (NS) therapy and final Disability Rating Scale (DRS) scores

After we excluded patients who died or were discharged before 7 days (*n* = 17), more patients in the early group had a favorable outcome (50.0% vs. 18.8%, *p* = 0.0011) compared to the late group. Final DRS scores (11.5 vs. 21, *p* = 0.0002) and GCS scores (14 vs. 10, *p* = 0.0018) were also better in the early NS group (Table 4). On bivariate analysis, there was a correlation between delaying NS therapy and worsening DRS scores (Fig. 1).

**Table 4:** Study outcomes for patients receiving neurostimulants (*n* = 121)

	Early (*n* = 52)	Late (*n* = 69)	*p*
*Cognitive outcome, n (%)*^*a*^			0.0011
Favorable	20 (50.0)	12 (18.8)	
Unfavorable	20 (50.0)	52 (81.2)	
Final DRS score, median (IQR)^a^	11.5 (7–20.8)	21 (15.3–24)	0.0002
Final GCS score, median (IQR)^a^	14 (11–15)	10 (7–14)	0.0018
Hospital LOS, median (IQR), d	21 (13–37)	32 (23–46)	0.0029
ICU LOS, median (IQR), d	12 (6–22)	22 (15–32)	< 0.0001
Mechanical ventilation duration, median (IQR), d	9 (4–18)	15 (9–24)	0.0003
Hospital free days, median (IQR)^b^	7 (0–15)	0 (0–5)	0.0011
ICU free days, median (IQR)^b^	16 (6–22)	6 (0–13)	< 0.0001
Mechanical ventilation free days, median (IQR)^b^	18 (10–24)	13 (4–19)	0.0003
*Medication-specific adverse events, n (%)*			
Seizures	2 (3.8)	6 (8.7)	0.4638
Tachyarrhythmia	1 (1.9)	3 (4.3)	0.6338
*Inpatient adverse events, n (%)*			
Acute kidney injury	5 (9.6)	11 (15.9)	0.4187
Bacteremia	5 (9.6)	10 (14.5)	0.5792
Cardiopulmonary arrest	4 (7.7)	5 (7.2)	1.0000
ICU readmission	3 (5.8)	5 (7.2)	1.0000
Pneumonia	21 (40.4)	39 (56.5)	0.0990
Urinary tract infection	3 (6.5)	2 (3.2)	0.6482
Venous thromboembolism	4 (7.7)	11 (15.9)	0.2652
*Discharge disposition, n (%)*			
Dead/hospice	10 (19.2)	5 (7.2)	0.0337
Home	6 (11.5)	3 (4.3)	
Inpatient rehabilitation	26 (50.0)	32 (46.4)	
LTACH/SNF	9 (17.3)	27 (39.1)	
Unknown/other	1 (1.9)	2 (2.9)	

DRS, Disability Rating Scale; GCS, Glasgow Coma Scale; ICU, intensive care unit; IQR, interquartile range; LOS, length of stay; LTACH, long-term acute care hospital; SNF, skilled nursing facility

^a^Excludes patients (*n* = 17) with a hospital LOS < 7 days or who died/were discharged to hospice

^b^Days free within 28 days

Among all patients in the NS subgroup (*n* = 121), hospital LOS (21 vs. 32 days, *p* < 0.0001), ICU LOS (12 vs. 22 days, *p* < 0.001), and mechanical ventilation duration (9 vs. 15 days, *p* = 0.0003) were significantly shorter in the early group. The early group also had more hospital, ICU, and mechanical ventilation free days. Complications were similar between groups, and the overall incidence of seizures (7.7%, *n* = 8) and tachyarrhythmias (3.8%, *n* = 4) was low. There was variation in discharge dispositions between NS groups (*p* = 0.0337); however, only LTACH/SNF discharge remained significantly different on pairwise comparison (early vs. late, 17.3% vs. 39.1%, *p* = 0.0099) (Table 4).

### NS Subgroup: Predicting Cognitive Impairment

We created a multivariable logistic regression model to predict a favorable cognitive outcome among patients receiving NS who survived and were not discharged before 7 days (*n* = 104). The variables selected a priori were entered into a backward stepwise regression model, and NS timing groups (early vs. late), Marshall score (V vs. I–IV), and ICU LOS remained in the final model (Table 5). Patients receiving early NS were three times as likely to have a favorable outcome compared to those receiving late NS (odds ratio 3.01, 95% confidence interval 1.11–8.18, *p* = 0.03). The overall model had good predictive ability (AUROC = 0.816).

**Table 5:** Predicting a favorable cognitive outcome among patients receiving NS

	Adjusted OR	95% CI	*p*
Early NS (vs. late)	3.01	1.11–8.18	0.031
ICU length of stay, d	0.93	0.89–0.98	0.004
Marshall score (V vs. I–IV)	0.20	0.06–0.65	0.0073

Excludes patients (*n* = 17) with a hospital length of stay < 7 days or who died/were discharged to hospice. Area under the receiver operating characteristic curve = 0.816

CI, confidence interval; ICU, intensive care unit; NS, neurostimulant; OR, odds ratio

## Discussion

In this multicenter study of patients with acute, severe TBI, approximately one third received NS. There was significant variation in the frequency of use of NS between hospitals, which is not surprising given the heterogeneity in ICU provider teams and differing approaches to TBI care across US trauma centers [32]. We found that patients prescribed NS had worse intracranial pathology, higher ISS, more NSIs, and more adverse events and experienced longer hospitalizations. These findings reflect how NS patients were overall sicker, likely explaining their worse cognitive status. Similarly, Morrison et al. found that children admitted to the ICU after TBI who received NS were more likely to have NSIs, be mechanically ventilated, and have significantly longer ICU and hospital LOS [33]. We believe that intensivists in our study may have given NS to these sicker patients as a salvage attempt to yield any glimpse of cognitive recovery. This theory could further be supported by our subgroup analysis finding that delaying NS initiation correlates with worsening final DRS scores.

Another plausible explanation for less favorable outcomes in the NS cohort is that a 12-day duration of NS is too brief to allow for a therapeutic effect. Many studies in the postacute setting have found that improvement in DRS scores occurred when patients received NS treatment for 4 to 6 weeks [3, 6, 7]. Similarly, although the goal of our study was to assess acute cognitive outcomes, a 28-day assessment may be too early to detect a meaningful clinical change. For example, McCrea et al. found that among patients with severe TBI, only 12.4% had a favorable cognitive outcome at 2 weeks, but this percentage increased to 45% at 3 months and 52.4% at 12 months [34]. Furthermore, DAI, which was more common in patients in our NS group, often requires an even longer time to demonstrate cognitive recovery [35, 36]. A longer treatment duration and assessment window may have been more revealing of the relationship between NS and cognition.

Nevertheless, when we performed our subgroup analysis of only patients receiving NS, we found that the early group was three times as likely to have a favorable outcome compared to the late group. The lower rates of tracheostomies and PEGs and more hospital and ICU free days in the early NS group could reflect a faster improvement in cognition because of NS, whereby decreasing the need for subsequent interventions. Alternatively, the early group may not have even needed these interventions because they were healthier and already beginning to emerge from a DoC. In this instance, clinicians may have chosen to prescribe NS to accelerate their recovery and improve their candidacy for TBI rehabilitation [37]. Regardless, our finding that early NS use was associated with more favorable cognitive outcomes is supported by a meta-analysis by Mohamed et al., who evaluated the impact of amantadine on patients with TBI [38]. They found a significant improvement in cognition when amantadine was given within the first week of injury. On meta-regression that included timing of treatment, patient age, and TBI severity, earlier administration of amantadine had the strongest cognitive effect [38].

In our NS subgroup analysis, we also observed more NSIs in the late group. It is possible that intensivists were reticent to initiate NS earlier in these patients due to the increased risk of posttraumatic seizures (PTS) seen in patients undergoing craniotomy [39, 40]. This concern is further compounded by amantadine’s theoretical potential to lower the seizure threshold [41]. Thus, among patients with NSIs, later NS prescribing may reflect these practice patterns. Interestingly, we found that PTS rates between the early and the late NS groups were similar and that the overall PTS incidence for the entire cohort was low. Rates of tachyarrhythmias were also similar between the two NS groups and were low for the entire cohort. These findings demonstrate that NS are safe to use during the inpatient setting and can be initiated within the first week of injury. We also believe that because there is such a low side effect profile, a trial of NS should be considered prior to definitive neurological prognostication and/or withdrawal of life-sustaining therapy.

Our study has several limitations. This study was not randomized, and the groups were not well matched. Had the groups been more similar, we may have been able to more accurately interpret the acute cognitive effect associated with NS. Second, NS prescribing was left to the intensivist’s discretion, and there was no standardization to how long patients received NS or when the drug was stopped. Although reflective of practice patterns across the United States, this lack of standardization introduces significant selection bias. Because these data were prospectively collected, it is also possible that study participation influenced providers to use NS more than they otherwise would.

In addition, although all patients had CT- or MRI-proven TBI, the classification of severe TBI was based on a GCS score ≤ 8. The GCS score is validated and ubiquitous in the setting of trauma, but a low presenting GCS score can be attributed to non-TBI pathology, such as substance intoxication, sedation, and/or neuromuscular blockades [42, 43]. Although we tried to overcome this issue by excluding patients who died or were discharged within 48 h, we still may have introduced sampling error.

This study was also limited by using the DRS and choosing 28 days as our end point. Although there is no singular tool to unequivocally measure cognitive function, other scales besides the DRS and GCS could have helped elucidate more granular cognitive details, such as sensory–perceptual–motor skills, memory, attention, and language [44]. Although the intent of this study was to explore NS use in the acute setting, restricting our assessments to 28 days is likely too brief of a time period to detect meaningful cognitive improvements. This limitation is compounded by the lack of data on NS use after discharge and other longitudinal assessments.

Cognitive function can also be influenced by several neuromodulating medications. Although we captured data on antipsychotic and propranolol use, we did not track other medications that could modulate neurocognition following a TBI, such as γ-aminobutyric acid agonists, selective serotonin reuptake inhibitors, and acetylcholinesterase inhibitors [5]. Finally, despite patients being appropriate candidates for certain discharge locations, the ultimate disposition can be influenced by socioeconomic factors and insurance status, which were not collected [45].

## Conclusions

In this multicenter study of patients with acute, severe TBI, nearly a third received NS. Patients receiving NS were more injured, had more procedural interventions, and had longer, more complicated hospital courses. Among individuals who received NS, treatment within 7 days was safe and was associated with favorable cognitive outcomes compared to later treatment. Future randomized studies are warranted to examine the effect of NS on cognitive outcomes in the acute setting.

## Supplementary Information

Below is the link to the electronic supplementary material.

Supplementary file1 (DOCX 17 KB)

Supplementary file2 (DOCX 30 KB)

Supplementary file3 (PDF 2066 KB)
