# Supplementary material for: Neurostimulant Use and Cognitive Outcomes in Patients with Acute, Severe Traumatic Brain Injury
Source: Neurocrit Care. 2025 Nov 5;44(2):486–95. doi: 10.1007/s12028-025-02400-3 (PMC13053455; doi:10.1007/s12028-025-02400-3)
Supplement: Supplementary file 1 — Supplementary file1 (DOCX 17 KB) [file 12028_2025_2400_MOESM1_ESM.docx]

**Appendix A:** Adverse outcome definitions

|  | |
| --- | --- |
| **AKI (acute kidney injury)** | Serum creatinine 3x baseline; **OR** an increase in serum creatinine to ≥ 4.0 mg/dl; **OR** the initiation of renal replacement therapy; **OR** urine output <0.3 ml/kg/h for > 24 hours  **exclude patients with renal failure prior to injury.* |
| **ARDS (acute respiratory distress syndrome)** | - Within 1 week of clinical insult - Bilateral opacities (not explained by effusions, collapse, nodules) - Respiratory failure (not explained by cardiac failure/fluid overload) - PaO_2_/FiO_2_ ≤ 300 mm Hg AND with a PEEP ≥ 5 |
| **Bacteremia** | A laboratory-confirmed bloodstream infection. |
| **Cardiopulmonary arrest** | Sudden cessation of cardiac activity with no normal breathing and no signs of circulation. |
| **DVT (deep venous thrombosis)** | Confirmed by venogram, ultrasound, or CT |
| **PE (pulmonary embolism)** | Confirmed by pulmonary arteriogram or CT angiogram |
| **Pneumonia [ventilator-associated]** | Mechanical ventilation for > 2 days **AND** diagnostic criteria for pneumonia. |
| **Tachyarrhythmia** | Abnormal heart rhythm with a ventricular rate of ≥ 100 bpm. |
| **Seizure** | Clinical picture **AND** supported by electroencephalogram data. |
| **Sepsis**  *either criteria acceptable | A documented infection **AND** ≥ 2 of the following:   - Temperature > 38° or <35° - Heart rate > 90 bpm - Respiratory rate > 20 breaths/min - WBC > 12,000 or <4,000 |
|  | A documented infection **AND** an acute increase of ≥2  Sequential [Sepsis-related] Organ Failure Assessment (SOFA) points. |
