# Supplementary material for: Neurostimulant Use and Cognitive Outcomes in Patients with Acute, Severe Traumatic Brain Injury
Source: Neurocrit Care. 2025 Nov 5;44(2):486–95. doi: 10.1007/s12028-025-02400-3 (PMC13053455; doi:10.1007/s12028-025-02400-3)
Supplement: Supplementary file 2 — Supplementary file2 (DOCX 30 KB) [file 12028_2025_2400_MOESM2_ESM.docx]

**Appendix B.** Distribution of neurostimulants across contributing sites.

*NS* neurostimulant; *UDL* upper decision limit; *LDL* lower decision limit
