# Supplementary material for: Neurostimulant Use and Cognitive Outcomes in Patients with Acute, Severe Traumatic Brain Injury
Source: Neurocrit Care. 2025 Nov 5;44(2):486–95. doi: 10.1007/s12028-025-02400-3 (PMC13053455; doi:10.1007/s12028-025-02400-3)
Supplement: Supplementary file 3 — Supplementary file3 (PDF 2066 KB) [file 12028_2025_2400_MOESM3_ESM.pdf]

EINSTEIN
Albert Einstein College of Medicine
Office of Human Research Affairs
Montefiore

Science at the heart of medicine
Institutional Review Board
Albert Einstein College of Medicine
FWA #00023382
Montefiore Medical Center
FWA #00002558
North Bronx Healthcare Network
FWA #00009807
Yeshiva University
FWA #00000140
White Plains Hospital
FWA #00023382

East Campus IRB
Jack and Pearl Resnick Campus
1300 Morris Park Ave., Belfer 1002
Bronx, NY 10461
718.430.2237 fax 718.430.8817

West Campus IRB
Montefiore Medical Center
3308 Rochambeau Avenue
Bronx, NY 10467
718.798.0406 fax 718.798.5657

# Notification of Renewal of Institutional Approval

**Date:** March 14, 2025

**Principal Investigator**: Anna Liveris, M.D.

| | | | |
|---|---|---|---|
| **Study Title:** | Cognitive Outcomes and NeuroStimulants among the Cerebrally Injured & Obtunded in the United States | | |
| **IRB #:** | 2021-12747 | **Reference #:** | 096845 |
| **Institutional Approval Date:** | 03/14/2025 | **Study Expiration Date:** | 03/13/2026 |

This is to inform you that the Office of Human Research Affairs (OHRA) has reviewed and extended the institutional approval period for the above referenced human research project for the period noted above.

**Expiration Notice**: Institutional approval for this study is limited to the period specified above. In order to gain re-approval, you must submit a Progress Report by 02/13/2026. To facilitate this, iRIS will send an email reminder 60 days prior to the due date. When this project is completed, submit a final Progress Report to close the file.

**To prevent lapses in institutional approval the OHRA recommends the following:**

- Submission of Progress Report in iRIS 6 weeks prior to the expiration date
- Setting up a calendar reminder to submit the Progress Report (automatic iRIS notifications are often sent to SPAM).
- Maintaining a list of COI disclosures and CITI training for all Key Personnel, and proactively updating the disclosures and training on a regular basis. We recommend that COI disclosures be updated on a quarterly basis.
- Notifying the PI by phone or email to sign off on the Progress Report submission (automatic iRIS notifications are often sent to SPAM).
- Proactively tracking the Progress Report submission in IRIS to make sure the it is progressing in the system in a timely manner. You may track the status of your submission by going to Study Assistant > My Studies > Click on the notepad > Click on the colored icon under "Track Location" (right hand side of the page) and note the location of the submission by the first row in the list.

<u>**Reminders**</u>

**Reportable Events** must be reported to the IRB in compliance with the Einstein IRB policy.

All changes to a study must receive IRB approval before they are implemented. The only exception to the requirement for prior IRB review and approval is when the changes are necessary to eliminate apparent immediate hazards to the subject (45 CFR 46.103(b)(4), 21 CFR 56.108(a)). In such cases, report the actions taken as a reportable event.

For a list of all currently approved documents, follow these steps: Go to Study Assistant – My Studies and open the study – Click on Informed Consent to obtain a list of approved consent documents and Other Study Documents for a list of other approved documents.

***Consent form posting requirement for federally sponsored clinical trials receiving initial approval from the IRB after January 20, 2019***
*The revised Common Rule requires that for each clinical trial conducted or supported by a Federal department or agency (such as the NIH), one IRB-approved informed consent form used to enroll subjects must be posted by the awardee on a publicly available Federal Web site.*

*The informed consent form must be posted on the Federal Web site after the clinical trial is closed to recruitment, and no later than 60 days after the last study visit by any subject, as required by the protocol.*

*At this time, there are two publicly available federal websites that will satisfy the consent form posting requirement: ClinicalTrials.gov and a docket folder on Regulations.gov (Docket ID: HHS-OPHS-2018-0021).*

If additional information is needed, please contact the Administrative Office at 718-430-2237.

# Institutional Review Board (IRB) Authorization Agreement

| | |
|---|---|
| **Institution Providing IRB Review (IRB of Record)** | The Ohio State University |
| **IRB of Record Registration #** | IRB00000294 |
| **IRB of Record Federalwide Assurance # (FWA)** | FWA00006378 |
| **Relying Institution** | Memorial Health University Medical Center |
| **Relying Institution's Federalwide Assurance #** | FWA00001689 |

| | | |
|---|---|---|
| The officials signing below agree that | Memorial Health University Medical Center | may rely on the designated IRB of Record for review and continuing oversight of its human subjects research described below |

**This agreement is limited to the following specific protocol(s):**

| | |
|---|---|
| **Name of Research Project** | Cognitive Outcomes and NeuroStimulants among the Cerebrally Injured & Obtunded in the United States: The CONSCIOUS Study |
| **Protocol Number** | Ohio State #2020H0365 |
| **Funding Agency (Award Number)** | N/A |

| **Point of Contact** | **Name** | **Phone** | **Email** |
|---|---|---|---|
| **Ohio State Primary Investigator** | Brett Tracy | | Brett.Tracy@osumc.edu |
| **Ohio State IRB Point of Contact** | Jess Mayercin-Johnson | 614-688-1059 | Mayercin-Johnson.1@osu.edu |
| **Relying Institution Co-Investigator** | Katherine McBride | 912-350-7412 | Katherine.McBride@hcahealthcare.com |
| **Relying Institution IRB Point of Contact** | Chableau Ford | 912-350-6866 | Chableau.Ford@hcahealthcare.com |

The review performed by The Ohio State University will meet the human subject protection requirements of the relying institution's OHRP-approved FWA. The IRB at Ohio State may serve as the HIPAA privacy board as necessary. The IRB at The Ohio State University will follow standard procedures for reporting its findings and actions to appropriate officials at the relying institution. Relevant minutes of IRB meetings will be made available to the relying institution upon request. The relying institution remains responsible for ensuring compliance with the IRB's determinations and with the Terms of its OHRP-approved FWA. This document must be kept on file by both parties and provided to OHRP upon request.

**Signature of Signatory Official of Relying Institution:**

| | |
|---|---|
| **Institution** | Memorial Health University Medical Center |
| **Signatory Official** | Mary Ann Beil, MTS |
| **Title** | VP, Corporate Ethics/Institutional Official |
| **Phone** | 912-350-5193 |
| **Email** | MaryAnn.Beil@HCAHealthcare.com |

Mary Ann Beil — 3/29/21

**Signature** **Date**

**Signature of Signatory Official of the Ohio State University (IRB of Record):**

| | |
|---|---|
| **Institution** | **The Ohio State University** |
| **Signatory Official** | Janet Weisenberger, PhD |
| **Title** | Senior Associate Vice President for Research |
| **Phone** | 614-247-4764 |
| **Email** | weisenberger.21@osu.edu |

DocuSigned by: Janet M. Weisenberger FD09B47EE7E64FC... — 03/31/2021

**Signature** **Date**

| | |
|---|---|
| **From:** | Buck-IRB |
| **To:** | tracy.210@osu.edu |
| **Cc:** | long.1694@osu.edu; Eggers, Christiane; Zottola, Kristina; mcdaniel.526@osu.edu |
| **Subject:** | Amendment #4 Approved for #2020H0365 |
| **Date:** | Monday, May 24, 2021 1:42:52 PM |

The Ohio State University

**Biomedical Sciences Institutional Review Board**

300 Research Administration building
1960 Kenny Road
Columbus, OH 43210-1063

orrp.osu.edu

05/24/2021

Study Number: 2020H0365
Study Title: Cognitive Outcomes and NeuroStimulants among the Cerebrally Injured & Obtunded in the United States: The CONSCIOUS Study

Type of Review: Amendment #4

Review Method: Expedited

Request to amend the research dated May 4, 2021 (add Jacobi Medical Center and Cooper Health as locations of research with IRB approvals for each; add OhioHealth as a relying site, external co-investigator Michelle Kincaid, M. Chance Spalding, and external key personnel Kimberly Sperwer, Jim Tsai, Josh Burton, Jason Hughes, and Stephanie Doris to the study team via SMART IRB IAA; increase number of Ohio State participants to 250 [150 at Ohio State, 50 at Memorial Health, and 50 at OhioHealth] and multi-site accrual goal to 750; revise protocol to reflect these changes).

Date of IRB Approval: 05/24/2021
Date of IRB Approval Expiration: 11/06/2021

Dear Brett Tracy,

The Ohio State Biomedical Sciences IRB **APPROVED** the above referenced research.

**Administrative Note:**

- As the university moves to a staged approach to restarting research activities, refer to Human Subjects Guidance and FAQs. If after reviewing this information and working through your college you have additional questions, please direct emails to research@osu.edu.

As Principal Investigator, you are responsible for ensuring that all individuals assisting in the conduct of the study are informed of their obligations for following the IRB-approved protocol

and applicable regulations, laws, and policies, including the obligation to report any problems or potential noncompliance with the requirements or determinations of the IRB. Changes to the research (e.g., recruitment procedures, advertisements, enrollment numbers, etc.) or informed consent process must be approved by the IRB before implemented, except where necessary to eliminate apparent immediate hazards to subjects.

This approval is issued under The Ohio State University's OHRP Federalwide Assurance #00006378 and is valid until the expiration date listed above. ***Without further review, IRB approval will no longer be in effect on the expiration date.*** To continue the study, a continuing review application must be approved before the expiration date to avoid a lapse in IRB approval and the need to stop all research activities. A final study report must be provided to the IRB once all research activities involving human subjects have ended.

Records relating to the research (including signed consent forms) must be retained and available for audit for at least 5 years after the study is closed. For more information, see university policies, Institutional Data and Research Data.

Human research protection program policies, procedures, and guidance can be found on the ORRP website.

Karla Zadnik, OD, PhD

Karla Zadnik, OD, PhD, Chair
Ohio State Biomedical Sciences IRB

AAHRPP

## Roden-Foreman, Jacob

**From:** IRB@mednet.swmed.edu
**Sent:** Wednesday, January 5, 2022 2:14 PM
**To:** Roden-Foreman, Jacob
**Subject:** ACTIVATED - Minimum approvals received

Sent Externally From: IRB@mednet.swmed.edu

Dear Study Team,

You are being notified because study # STU-2020-1292 [eresearch.swmed.edu], titled, Cognitive Outcomes and NeuroStimulants among the Cerebrally Injured & Obtunded in the United States: The CONSCIOUS Study, has been approved by the IRB.

On Wednesday, January 5, 2022, the study met all the required approvals and may begin at the below performance sites:

| Approval Component | Component Name | Status |
|---|---|---|
| Performance Site | UTSW | Not Applicable |
| Performance Site | Children's | Not Applicable |
| Performance Site | Parkland | Not Applicable |
| Performance Site | THR | Approved |
| Coverage Analysis | Coverage Analysis | Not Required |
| Contract | Clinical Trial Agreement | CTA Not Required |

**Approval by the appropriate authority at each performance site is required before subjects may be enrolled at that site. If the study is sponsored, sponsor activation may be required before study activities may begin.**

Thank you,

Warning: This is a private message for authorized UT Southwestern employees only. If the reader of this message is not the intended recipient you are hereby notified that any dissemination, distribution or copying of this information is STRICTLY PROHIBITED.

University of Texas Southwestern Medical Center
Institutional Review Board

5323 Harry Hines Boulevard
Dallas, Texas 75390-8843
phone: 214-648-3060
fax: 214-648-2171

THE OHIO STATE UNIVERSITY

**Biomedical Sciences Institutional Review Board**

300 Research Administration building
1960 Kenny Road
Columbus, OH 43210-1063

orrp.osu.edu

11/06/2020

Study Number: 2020H0365
Study Title: Cognitive Outcomes and NeuroStimulants among the Cerebrally Injured & Obtunded in the United States: The CONSCIOUS Study

Type of Review: Initial Submission

Review Method: Convened

Date of IRB Approval: 11/06/2020
Date of IRB Approval Expiration: 11/06/2021

Dear Brett Tracy,

The Ohio State Biomedical Sciences IRB **APPROVED** the above referenced research.

In addition, the following were also approved for this study:

- Adults with impaired decision-making ability
- Waiver of Consent Process
- Consent by Legally Authorized Representative
- Full Waiver of HIPAA Research Authorization

**Administrative Note:** The Biomedical IRB determined that this research presents no more than minimal risk to participants. When submitting applications for continuing review, please request expedited review.

**Administrative Note:**

- As the university moves to a staged approach to restarting research activities, refer to Human Subjects Guidance and FAQs. If after reviewing this information and working through your college you have additional questions, please direct emails to research@osu.edu.

As Principal Investigator, you are responsible for ensuring that all individuals assisting in the conduct of the study are informed of their obligations for following the IRB-approved protocol and applicable regulations, laws, and policies, including the obligation to report any problems or potential noncompliance with the requirements or determinations of the IRB. Changes to the research (e.g., recruitment procedures, advertisements, enrollment numbers, etc.) or informed consent process must be approved by the IRB before implemented, except where necessary to eliminate apparent immediate hazards to subjects.

This approval is issued under The Ohio State University's OHRP Federalwide Assurance #00006378 and is valid until the expiration date listed above. ***Without further review, IRB approval will no longer be in effect on the expiration date.*** To continue the study, a continuing review application must be approved before the expiration date to avoid a lapse in IRB approval and the need to stop all research activities. A final study report must be provided to the IRB once all research activities involving human subjects have ended.

Records relating to the research (including signed consent forms) must be retained and available for audit for at least 5 years after the study is closed. For more information, see university policies, Institutional Data and Research Data.

Human research protection program policies, procedures, and guidance can be found on the ORRP website.

Karla Zadnik, OD, PhD, Chair
Ohio State Biomedical Sciences IRB

LOMA LINDA UNIVERSITY
HEALTH

**INSTITUTIONAL REVIEW BOARD**
***HUMAN RESEARCH & COMPLIANCE***
*24887 Taylor Street • Suite 201 • Loma Linda, CA 92350*
*(909) 558-4531 (voice) • (909) 558-0131 (fax)*

## *Initial Approval Notice - Expedited* — *IRB# 5210173*

To: **Mukherjee, Kaushik**
Department: **SM: Surgery**
Protocol: **Cognitive Outcomes and NeuroStimulants among the Cerebrally Injured & Obtunded in the United States: The CONSCIOUS Study**

This study was reviewed and approved administratively on behalf of the IRB. This decision includes the following determinations:

Risk to research subjects: **Minimal**
Approval begins: **07-May-2021**
Stipulations of approval: **Informed Consent waived per 45 CFR 46.116(d) and HIPAA authorization waived per 45 CFR 164.512 (i)(2)(ii).**
See attached list of items (if applicable).
See Appendix A for Conditions of Approval.

Adverse events and unanticipated problems must be reported in accord with the attached Adverse Event Reporting Matrix **A.**

All investigators are responsible for assuring that studies are conducted according to the approved protocol. Principal investigators are responsible for the actions of sub-investigators and staff with regard to this approval.

Please note the PI's name and the assigned IRB number, as indicated above, on any future communications with the IRB.

Direct all communications to the IRB c/o Human Research and Compliance.

Thank you for your cooperation in LLUH's shared responsibility for the ethical use of human subject in research.

IRB Chair/Designee

05/07/2021
Date

Loma Linda University Health holds Federalwide Assurance (FWA) No. 00006447 with the U.S. Office for Human Research Protections and the IRB registration no. is IORG0000226. This Assurance applies to the following: Loma Linda University, Loma Linda University Medical Center (including Loma Linda University Children's Hospital, LLUMC East Campus Hospital), Loma Linda University Behavioral Medicine, and affiliated medical practices groups.

**IRB Chair:**
Andrea Ray, MD
*Department of Plastic Surgery*
(909) 558-4531 • irbchair@llu.edu

**Executive Director**
Amy L. Casey, MBA
*Human Research & Compliance*
Ext 14658 • acasey@llu.edu

LOMA LINDA
UNIVERSITY
HEALTH

# CONDITIONS OF IRB APPROVAL

*IRB REQUIREMENTS FOR IMPLEMENTING FEDERAL AND INSTITUTIONAL IRB POLICIES*

## General

1. No subjects may be involved in any study procedure prior to the date indicated in the IRB approval notice or after the expiration date. This includes any testing solely to determine eligibility.
2. Unless IRB has stipulated a waiver of informed consent, the current IRB-approved consent form (with stamp) must be used to enroll subjects. The official consent accompanying this letter must be used as the master for making copies to provide to prospective study participants.
3. All recruitment materials and methods must be approved by the IRB prior to being used.
4. All modifications (e.g. changes to the protocol, study personnel, the informed consent document(s)/process, or number of subjects) must be IRB approved prior to implementation (see http://research.llu.edu/changerequest.asp)
5. No study activities may be conducted after the IRB approval end date unless determined to be medically necessary by the PI.
6. To avoid disruption of a study, the PI should request renewal of IRB approval by promptly submitting a research report form (see http://research.llu.edu/researchreport.asp)

## Records retention

### Minimum requirements

- All records relating to this project, including signed consent forms, must be kept on file for at least 3 years following completion of the study, unless special conditions apply (see below).
- Study records MUST remain at the institution under the jurisdiction of a responsible party to be able to locate records after study closure. This includes approved IRB documents, as well as case-report forms, tapes, or transcripts, and all other data collection instruments and source documents.

### Other special conditions

- Records involving the generation, disclosure, and/or use of Protected Health Information (PHI) should be retained for six years.
- Minors in research: records must be retained for seven years after all minors enrolled in the study reach the age of majority [age 18 in California]
- Records pertaining to in vitro fertilization studies or research involving pregnant women must be retained for a total of 25 years after study closure.
- Records generated from sponsored clinical trials may not be disposed until written permission is obtained from the sponsor.

### FDA-regulated studies - In addition to minimum requirements:

- For drugs and devices with approved marketing applications, the retention period is two years after FDA approval.
- For drugs and devices where no application is filed or the application is not approved, the retention period is two years after the investigation is discontinued and FDA is notified. 21 CFR 312.57 and 21 CFR 812.140.

ver. 8/1/2014

## A: Non-FDA *without* DMC

# ☞*Internal Event*

**IRB *itself* Performs AE Oversight**
**MINIMAL RISK STUDY**

(Include shaded boxes for studies **With** Federal Funding)

| BY Whom | TO Whom | WHAT KIND | WHEN |
|---|---|---|---|
| **Investigator** | IRB | **1, 2**<br>SERIOUS<br>RELATED (1) | Within<br>5 working days of<br>investigator awareness<br>OHRP Guidance<br>45 CFR 46.103 |
| | | **5**<br>NOT SERIOUS<br>RELATED<br>UNEXPECTED | Within 10 working days<br>of investigator awareness<br>OHRP Guidance<br>45 CFR 46.103 |
| **IRB** | Institutional Official | **1, 5**<br>RELATED<br>UNEXPECTED<br>that suggest study risks are greater than previously recognized | Within 10 working days<br>of notice to IRB<br>Institutionally required reporting time (2) |
| **Institutional Official** | OHRP<br>and<br>Funding Agency | | Within one month of notice to IRB<br>OHRP Guidance<br>45 CFR 46.103 |

(1) Any serious and related AE that occurs on a study correctly ranked as minimal risk is unexpected, by definition.

(2) To allow time for I/O review before reporting to OHRP

## A & B *without* DMC ☞ ***Unanticipated Problems***
### IRB *itself* Performs AE Oversight

(if **With** Federal Funding – add shaded boxes)

| BY Whom | TO Whom | WHAT KIND<br>(AE or ADR) | WHEN |
|---|---|---|---|
| **Investigator *** | IRB | **1**<br>SERIOUS<br>RELATED<br>that are<br>UNEXPECTED<br>and<br>that suggest the study risks are greater than previously recognized | Within 5 working days<br>of investigator awareness<br>(OHRP guidance:<br>Dated 01-15-07<br>V.(2) ) |
| | | **5**<br>NOT SERIOUS<br>RELATED<br>that are<br>UNEXPECTED<br>and<br>that suggest the study risks are greater than previously recognized | Within 10 working days<br>of investigator awareness<br>(OHRP guidance:<br>Dated 01-15-07<br>V.(2) ) |
| **IRB** | Institutional Official | **1, 5**<br>RELATED<br>that are<br>UNEXPECTED<br>and<br>that suggest the study risks are greater than previously recognized | Within 10 working days<br>of notice to IRB<br>(not regulatory- this allows time for I/O to consider before reporting to OHRP) |
| **Institutional Official** | OHRP<br>and<br>Funding Agency | | Within one month of notice to IRB<br>(OHRP Guidance) |

LOMA LINDA UNIVERSITY
Research Affairs

September 1, 2019

To Whom It May Concern,

The Department of Health and Human Services issued revisions to the regulations governing human subject research. As a result, Continuing Review is no longer required for most studies that qualify for the expedited review process unless the IRB has a reason to require it and can justify that reason. These apply to studies that have an **initial approval date** on or after January 21, 2019. This change means:

- No study end date will be reflected on your IRB approval letter
- No end date will be reflected on your informed consent

Even when continuing review is not required for the project, the PI must still:

- Submit amendments for projects changes including changes to personnel
- Report Adverse Events as required
- Terminate the project with the IRB once it ends

If you should have any questions or concerns, please do not hesitate to contact me.

Sincerely,

Amy L. Casey, MBA
Director, Human Research and Compliance
IRB Administrator

*A Seventh-day Adventist Organization*
LOMA LINDA UNIVERSITY HEALTH | Human Research & Compliance
24887 Taylor Street, Suite 201, Loma Linda, California 92354
909-558-4831

**Attachments:**
- Expedited Review Approved by Chair with Waiver of Informed Consent.pdf

## *Cooper Health IRB* — *Expedited Approval Notification*

To: TANYA EGODAGE
From: IRBOnlineHelp@cooperhealth.edu
Subject: Protocol #21-026
Date: 02/08/2021

The protocol, **Cognitive Outcomes and NeuroStimulants among the Cerebrally Injured & Obtunded in the United States: The CONSCIOUS Study,** has been approved by, or on behalf of the IRB Chair under 45 CFR 46.110 on **02/08/2021**.

(5) Research involving materials (data, documents, records, or specimens) that have been collected, or will be collected solely for nonresearch purposes

The approval of your study is valid through 02/07/2022.

**Data Use Agreement (DUA) Pending**
The DUA must be finalized prior to sharing PHI outside of Cooper. Please submit an amendment with the finalized DUA once it has been completed. **You are not authorized to share data containing PHI until the DUA is completed and the IRB amendment is approved.**

Approved/Acknowledged Files:

| | |
|---|---|
| **Application Form** | 01/23/2021 Study Protocol (approved).docx |
| **Pre-Study Questionnaire** | 01/23/2021 Pre-Protocol Questionnaire.pdf |
| **Additional Resources** | 01/23/2021 IRB CONSCIOUS study approval.pdf |
| **Pre-Review Response Memo** | 02/02/2021 pre-review comments with responses.docx<br>02/06/2021 board member review with comments.docx |
| **Data Collection Form** | 01/23/2021 CONSCIOUS study data dictionary & collection tool.pdf<br>01/25/2021 CONSCIOUSSubjectIDAndFollowUpLog.pdf |

This study has been approved for 20 subjects. In order to enroll more than this number, please submit an amendment for review by the IRB.

The IRB approved this study for the use of substituted consent for subjects who lack the capacity to consent on their own behalf according to the New Jersey Access to Medical Research Act (N.J.S.A 26:14.1 - 14.5).

**Waiver/Alteration of Informed Consent:**
A waiver of informed consent has been approved by the IRB per the regulations at 45 CFR 46.116(d).

**HIPAA:**
This protocol is approved for waiver of HIPAA Authorization for all subjects according to 45 CFR 164.512(i)(2)(ii).

**Continuing Review/Study Closure:**
At Cooper, research meeting the Expedited criteria must be reviewed by the IRB at least every 365 days. You will receive electronic reminders about your upcoming expiration date beginning 8 weeks prior to the expiration of this project's approval. If you wish to request another year of approval, please submit your Continuing Review submission by **01/10/2022.** If the research study is not re-approved before the expiration date, accrual of new subjects (including review of medical records and all data analysis) must be suspended until IRB approval has been renewed.

If during your approval period or at the time of the study's expiration you are done with all research related activities, including analysis of your data, please submit a Study Closure Report to the IRB.

**Amendments:**
As the Principal Investigator, it is your responsibility to inform the IRB of any and all changes to the approved study, including but not limited to changes in investigators and study staff and revisions to the protocol and/or consent form. You must have IRB approval before initiating any changes to the study except changes that are necessary to eliminate immediate hazards to the subjects.

**Unanticipated Risk Reporting:**
The Principal Investigator must immediately report to the Cooper IRB any unanticipated problems involving risks to subjects or others and any serious harm to subjects.

The Cooper IRB

COOK COUNTY HEALTH

Leadership
Toni Preckwinkle
President
Cook County Board of Commissioners
Israel Rocha, Jr.
Chief Executive Officer
Cook County Health

Board of Directors
M. Hill Hammock
Chair of the Board
David Ernesto Munar
Vice Chair of the Board
Robert Currie
Hon. Dr. Dennis Deer, LCPC, CCFC
Mary Driscoll, RN, MPH
Raul Garza
Ada Mary Gugenheim
Joseph M. Harrington
Mike Koetting
Heather M. Prendergast, MD, MS, MPH
Robert G. Reiter, Jr.
Otis L. Story, Sr.

October 19, 2022

William Brigode, MD
1950 W. Polk St
Chicago, IL 60612

**RE: Study 21-086X** Cognitive Outcomes and NeuroStimulants among the Cerebrally Injured & Obtunded in the United States

Dear Dr. Brigode:

Thank you for submitting your application for exemption to the Cook County Health Institutional Review Board (CCH IRB). This is to inform you that the above referenced Study was examined in light of the federal regulations that govern the protection of human subjects. The CCH IRB has determined your Study meets the criteria set forth in 45CFR46.104 for exemption from IRB review. Please note that IRB consideration does not substitute for departmental clearance.

**Documents Submitted:** Tentative DUA, Executive DUA, Data Collection Tool

**Exempt Category:** Exempt Category 4: Secondary research uses of identifiable private information or identifiable biospecimens.

**HIPAA Waiver:** Yes
**Expiration Date**: None/Exempt

Even though your project is exempt from IRB review, the research must be conducted according to the proposal submitted to the CCH IRB. If changes to the approved protocol occur, a revised protocol must be reviewed and approved by the IRB before implementation for determination of whether the study remains exempt.

As the Principal Investigator, you still have ultimate responsibility for the ethical conduct of the research and for the protection of the rights and welfare of all human subjects involved in the research, in accordance with The Belmont Principles.

Sincerely,

Audrean LaGrone

Audrean LaGrone
IRB Manager, Research & Regulatory Affairs
